# The Effect of Supplementation of Fish Protein Hydrolysate to the BSF-Based Aquafeed on the Growth, Survival, Fatty Acids, and Histopathology of Juvenile Lobster (*Panulirus ornatus*)

**DOI:** 10.1155/2024/8579991

**Published:** 2024-06-25

**Authors:** Ishaaq Saputra, Yih Nin Lee, Ravi Fotedar

**Affiliations:** ^1^ Faculty of Engineering and Sciences Curtin University, CDT 250, Miri Sarawak 98009, Malaysia; ^2^ School of Molecular and Life Sciences Curtin University, Kent Street, Bentley 6102, WA, Australia

## Abstract

The present study aims to evaluate the effect of liquid fish protein hydrolysate (FPH) following fishmeal substitution with full-fat and defatted BSF (black soldier fly, *Hermetia illucens*) meal in the feeds of juvenile ornate spiny lobster, *Panulirus ornatus*. The physiological aspects of juvenile lobsters including growth, fatty acids profile, and histopathology were observed. Six isoenergetic experimental feeds having a protein-to-energy ratio of 26 CP mg kJ^−1^ were formulated with the substitution of fishmeal at 25% using liquid FPH, full-fat BSF (FBSF), defatted BSF (DBSF), and their combination. The specific growth rate, final body weight, final total length, and length increment of juvenile lobsters (initial weight was 0.21 ± 0.01 g and total length was 20.53 ± 0.12 mm) were significantly affected by the fishmeal substitution (*P*  < 0.05) and improved with the addition of liquid FPH in the feeds containing FBSF and DBSF. The whole body proximate analysis showed that the liquid FPH to the feeds containing DBSF increased the ash and protein content significantly (*P*  < 0.05). The total monounsaturated fatty acids (∑MUFA), saturated fatty acids (∑SFA), and omega 9 fatty acids (∑*n*−9 FA) of juvenile lobsters' whole bodies fed with dietary DBSF and FPH supplementation were significantly higher than those of others (*P*  < 0.05). The histopathological analysis indicated that the villus size and the muscle thickness in the intestine were not significantly affected by FPH supplementation. However, the hepatopancreas histopathology indicated the presence of B-cells and R-cells in the juvenile lobsters fed with FPH-supplemented feeds. The present results suggested the supplementation of liquid FPH to the formulated feed with FBSF and DBSF for juvenile lobsters can improve the lobsters' growth and fatty acids availability.

## 1. Introduction

The ornate spiny lobster (*Panulirus ornatus*) remains a high-value fisheries commodity that has been traded throughout the globe. With an annual value of 5 BN $, it is the most expensive among crustacean commodities [1]. Inhabiting the coastal area in the trophic region [2], *P. ornatus* undergoes a complex life cycle with a significant metamorphosis phase to a juvenile lobster [3]. Despite the valuable potential market, the exploitation of *P. ornatus* should be maintained to sustain the lobster fishing industry. The complete hatchery-based larvae production of *P. ornatus* has been reported for research purpose [4] and pilot commercial operations (ornatas.com.au). The production of lobster larvae in a hatchery setting is currently limited for traditional lobster farmers and is a substantial obstacle to lobster aquaculture. In contrast, lobster aquaculture by collecting seed stock (puerulus or early juveniles) from the wild has been practiced since the 1990s in major lobster-producing countries, such as Vietnam and Indonesia [5, 6, 7]. The primary feed sources for lobster aquaculture are trash fish, clams, or other bivalves [8]. While these natural feeds provide adequate nutrition requirements for lobster growth [9], this feeding practice poses issues in environmental sustainability. Therefore, developing formulated feeds for lobsters is essential to promote sustainable lobster aquaculture industry [10].

The black soldier fly (BSF) (*Hermetia illucens*), henceforth abbreviated as BSF, is one of insects that is suitable aquafeeds for shrimp [11, 12, 13]. The nutrition of BSF is influenced by different substrates provided during their rearing [14]; BSF reared in organic waste has a higher fat than BSF reared in industrial waste. Aquaculture generally uses two types of BSF meal: defatted BSF (DBSF) and full-fat BSF (FBSF). FBSF is largely unprocessed and contains all fats up to 400 g kg^−1^ dry weight [15, 16], which can negatively impact the growth of fish or crustaceans. DBSF undergoes a fat removal process [17] either using mechanical compression or chemical extraction, which, according to [16, 17, 18], influence the composition of the final DBSF product. Both types of BSF have been used in several crustacean feeding experiments with mixed results, including [11] who stated that the suboptimum growth and immunity response of *Penaeus vannamei* after BSF meal inclusion in the diet may be associated with BSF low digestibility.

Protein hydrolysate can be used as a sole protein source or as a supplement in feed formulation. It has been extensively reported that protein hydrolysate can improve the digestibility of some feed ingredients and provide the optimization of growth results and immunological aspects of crustaceans [19, 20, 21, 22, 23, 24, 25], and increase the efficacy of non-fishmeal ingredients in the feed [26, 27, 28, 29, 30, 31, 32]. While protein hydrolysate from squid does not influence the growth of *P. vannamei* [25, 33, 34], protein hydrolysate has improved the survival rate, growth, and immunity of other crustaceans species such as tiger prawn, *Penaeus monodon* fed dietary plant-based protein diet [30, 31]. It brings new hope to use fish protein hydrolysate (FPH) in aquafeed production.

A previous study by Saputra and Fotedar [35] has reported that supplementing up to 35% DBSF meal in a formulated feed for juvenile lobsters does not impose negative impact on the physiological response of the species but unfortunately, even at the most significant level of supplementation, the optimum growth and survival of juvenile lobsters were still lower than those of other studies [4, 36]. Accordingly, it is crucial to employ additional feed supplements such as protein hydrolysate to improve the absorption of BSF nutrition as a non-fishmeal ingredient. Several studies have claimed the success of FPH in improving the growth and immunology of crustaceans, but little is known about its effect on lobster feeds. The present study evaluates the supplementation of protein hydrolysate using FBSF meal and DBSF meal as protein sources for juvenile lobsters.

## 2. Materials and Methods

### 2.1. Experimental Design

A total of 200 early juvenile lobsters *P. ornatus* (initial weight 0.19 ± 0.01 g, initial length 20.13 ± 0.12 mm) were purchased from a local fisherman in Lombok, Indonesia. Juvenile lobsters were packed in oxygenated plastic bags filled with 100 mL 30 ppt seawater (10 lobsters per bag). Upon arrival, all juvenile lobsters were acclimated in a 180 L container box equipped with net cuts (50 cm × 25 cm), 60 pipe cuts (Ø 100 mm, 4 cm), and an aeration system. After 3 days of the acclimation period, 180 early-stage juvenile lobsters were stocked into 18 tanks of 60 L aquaria (10 lobster per aquarium) equipped with individual filtration systems, hides made of pipe and net cuts, and a designated feeding area made of polystyrene (16 cm × 11 cm × 0.3 cm) glued to the aquarium bed using silicone sealant.

### 2.2. Feeding Experiment

The juvenile lobsters were kept for 8 weeks of feeding experiment. In this study, the fishmeal ingredients were sourced from bycatch *Nemipterus virgatus* with crude protein content of 56%. In this study, the protein-to-energy ratio of 26 mg kJ^−1^ of the feed was used as previously reported by Saputra and Fotedar [37]. Six experimental feeds were formulated using Feed LIVE software version 1.52 from Live Informatics Company Limited (Thailand) and supplemented with approximately 25% of either FPH, FBSF, DBSF, or the combination of three (Table 1). The six experimental feeds were T1 (fishmeal), T2 (25% FPH), T3 (25% FBSF), T4 (25% DBSF), T5 (25% FPH and 25% DBSF), and T6 (25% FPH and 25% FBSF). The fatty acids composition of the BSF and FBSF as feed ingredients is presented in Table 2 [16]. The amino acid composition of FM and DBSF [35] and FBSF [16] is presented in Table 3. All ingredients in each treatment were mixed in an automatic food processor (Kawano 250G, China), then added with a little water to form a dough. The dough was then processed using a food mincer (Oxone OX861n, Indonesia) until fine granules were formed, then stored in a sealed plastic bag and kept in a dry place until used as moist diets.

The experimental feed was given three times a day at 15% wet body weight and uneaten feed was removed from the feeding area. The room condition for the experiment was maintained at 29−30°C, with the light intensity of 12 hr day : 12 hr dark and the water salinity of 29.44–0.73 ppt.

### 2.3. Feed Proximate Composition Analysis

The proximate composition including protein, lipid, carbohydrate, ash, and moisture content was determined. The protein content was measured using a copper catalyst and boric acid according to an established protocol [38], while the lipid content was determined using combustion method according to Association of Official Agricultural Chemists [39]. To determine the ash content, the gravimetry method was used by placing 2–6 g of each experimental feed in crucibles and then heating it in a furnace at 550°C for 4 hr until the ashing process was completed. The moisture content of the feed was determined using the gravimetry method where 1–3 g of sample was heated in the oven at 105°C for 3 hr, then weighed. The heating process was deemed completed when the samples reached the constant weight. Then, the moisture content was calculated by subtracting the initial weight of sample-filled crucible from the final weight of sample-filled crucible and then dividing it by the initial weight of sample-filled crucible (W−W1)/W. The total carbohydrate content was determined using difference method by subtraction of 100% by the cumulative value of crude protein, fat, moisture, and ash. The fatty acids content was analyzed using gas chromatography method as described by Ratnayake et al. [40] where the peak of chromatograph was determined by comparing the retention time of every fatty acid component in the samples to that in fatty acid reference.

### 2.4. Intestine and Hepatopancreas Histopathology

At the end of the feeding trial, the juvenile lobsters from each treatment were collected and stored in Davidson solution for 24 hr before being subjected to histopathological analysis. The intestine (midgut) and hepatopancreas of juvenile lobsters were collected using the hematoxylin–eosin staining according to our previous study [35]. Overall, a total of six pathology sections were observed using a light microscope (Carl Zeiss Primo Star, US).

### 2.5. Data Collection and Calculation

At the initial feeding experiment, the mean values of wet body weight, total length, and carapace length were measured as the initial data. At the final stage of experiment, the effects of the treatment were measured from five biological parameters as follows:

1. Survival rate (%) = 100 × (final number of lobster/initial number of lobster).
2. Weight gain (%) = 100 × (final wet weight (g) – initial wet weight (g)).
3. Specific growth rate (% body weight/day) = 100 × (ln final wet weight (g) – ln initial wet weight (g))/days.
4. Molting rate = 100 × (number of molted lobster/total number of lobster).
5. Length increment = 100 × (final body length − initial body length)/final body length.

### 2.6. Statistical Analysis

All data were analyzed using SPSS for Windows version 25.0 (IBM, New York, USA). The effects of fishmeal replacement with DBSF, FBSF, FPH, and their combination on specific growth rate, total length increment, carapace length increment, and molting rate of the lobster juvenile were examined using a one-way ANOVA analysis with post hoc Tukey's HSD multiple comparison tests. The statistical significance was evaluated at *P*  < 0.05.

## 3. Results

The production performance of juvenile lobsters fed on FBSF and defatted black soldier fly meal (DBSF) with and without the supplementation of FPH is presented in Table 4. The results showed no significant differences in the mean survival rate and molting rate of juvenile lobsters across treatments at the end of the feeding experiment. The survival rate of juvenile lobster ranged from 50.0% ± 0.07% to 66.7% ± 0.04% while the molting rate ranged from 62.50% ± 0.13% to 79.17% ± 0.04%. In contrast, the final body weight, total length, specific growth rate, and length increment of juvenile lobsters fed on diet containing DBSF plus FPH supplementation were significantly higher.

The nutritional composition of juvenile lobsters is presented in Table 5. The crude protein content of juvenile lobster fed the combination of FBSF and FPH (T6) was significantly different (*P*  < 0.05). The highest crude protein content was found in the juvenile lobsters fed with FBSF plus FPH supplementation (T6) while the significantly higher (*P*  < 0.05) fat content was observed in juvenile lobsters fed with FBSF without FPH supplementation (T3). In contrast, the lowest carbohydrate was observed in T1, T3, and T6.

The fatty acid analysis of experimental feeds revealed that linolenic acid, Eicosatrienoic, and erucic acid were found only in the feed containing fishmeal included as the protein source. Meanwhile, the arachidic and eicosadienoic acids were found in fishmeal and FBSF feed (Table 6). In addition, the feed with FBSF has a significantly higher content of saturated fatty acid (SFA), unsaturated fatty acid, polyunsaturated fatty acid (PUFA), monounsaturated fatty acid (MUFA), omega 3 fatty acids (*n*−3 FA), omega 6 fatty acids (*n*−6 FA), and omega 9 fatty acids (*n*−9 FA).

The fatty acids analysis of the juvenile lobster's whole body indicated several fatty acids including palmitic, palmitoleic, stearic, oleic, and linoleic acids. Those fatty acid compounds were significantly higher (*P*  < 0.05) in juvenile lobsters fed with FBSF than the other treatments, and palmitoleic was only found in juvenile lobsters fed with diets supplemented with non-fish protein hydrolysate (Table 7).

**Figure 1:**
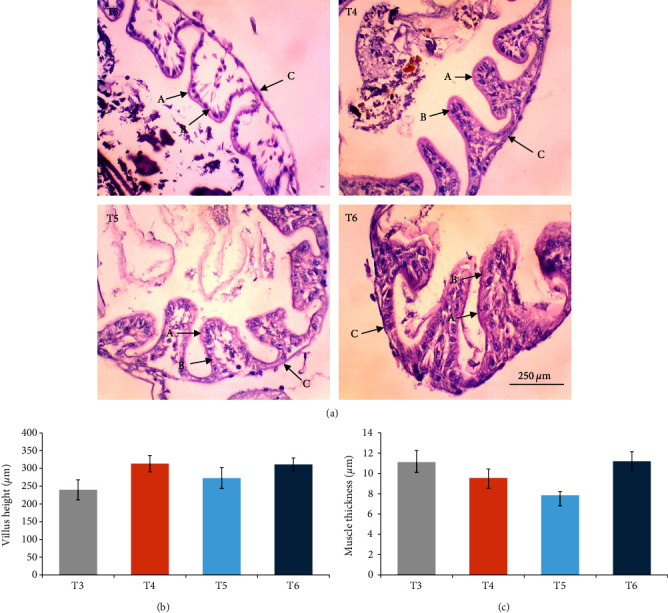
Effects of dietary fish protein hydrolysate, defatted black soldier fly, full-fat black soldier fly, and their combination on intestine morphology of juvenile lobsters (*P. ornatus*) at 400x magnification. (a) Intestinal histopathology variation by hematoxylin and eosin staining; (A) intestinal peritrophic membrane; (B) epithelial cells; and (C) muscle layer. (b) The villus height of the intestinal tissue. (c) The thickness of the muscle layer in the intestinal tissues.

**Figure 2:**
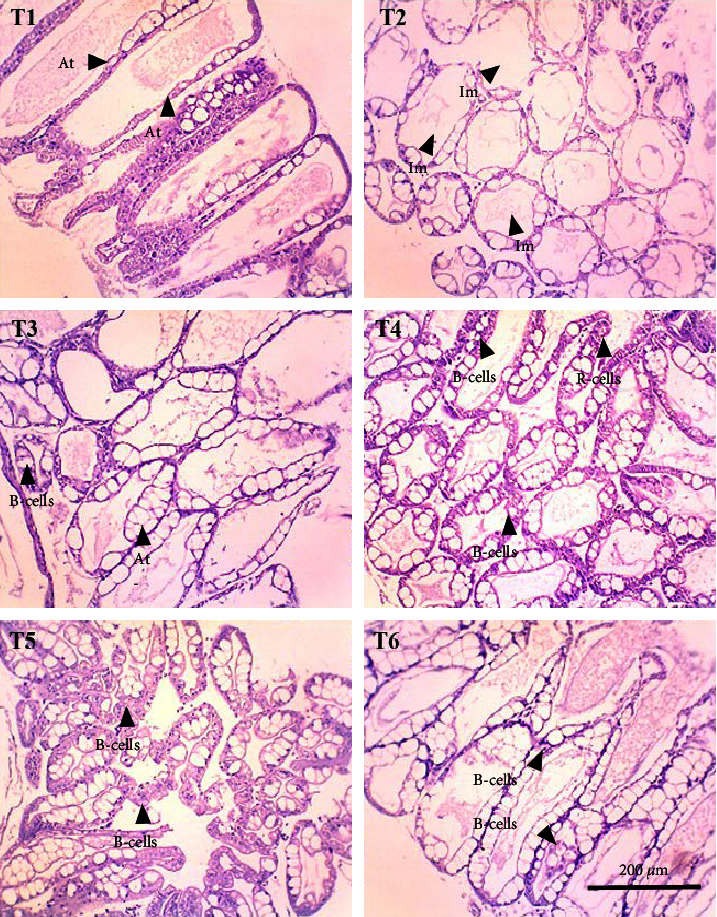
The histopathology of hepatopancreatic tubules in longitudinal and transverse section juvenile lobsters at 100x magnification (At = atrophy, Im = inflammation).

In this study, the midgut histopathology of juvenile lobsters from T1 and T2 could not be generated due to damaged samples. There were no significant differences in the mean of villus height and muscle thickness in lobsters' midgut across treatments (*P*  > 0.05). The degeneration of submucosal occurred in the midgut of juvenile lobsters in T4 (Figure 1). The hepatopancreas histopathology showed atrophy on the tubules of juvenile lobsters fed fishmeal, FPH, and DBSF. The hemocyte infiltration was found in juvenile lobsters fed with FPH. While B and R-cells were active in the hepatopancreas of juvenile lobsters fed DBSF, the B-cell was found in hepatopancreas of juvenile lobsters fed with FBSF plus FPH supplementation (Figure 2).

## 4. Discussion

The present study has shown that the supplementation of FPH significantly influences the most physiological response of juvenile lobsters. At the supplementation level of 25%, FPH improves the final total length and specific growth rate. While previous study reported the effectiveness of fish protein hydrolysate supplementation on crustaceans [19, 21, 22, 25, 41], this study contributes a specific finding: an improved absorption of FPH nutrition, which ultimately promotes the growth of juvenile lobsters, despite the suppressed growth rate when compared to other studies [4, 42]. The lower growth rate in this study is probably due to different sources of juvenile lobsters and feed formulations. The role of FPH supplementation is significant in FBSF-fortified feeds, resulting in improved growth rate and final body weight of juvenile lobsters. The most concerning issue related to the utilization of FBSF in aquafeed is the high lipid content, which potentially causes negative effects on fish. However, this study has shown that FPH can enhance the nutrition efficacy of FBSF. FPH has been reported to play an important role in providing functional and antioxidant protein for crustacean [43, 44]. A claim that FPH supplemented into crustacean feed does not significantly affect the crustacean's growth and survival of the animal [25, 45] is challenged by other studies reporting the opposite results [19, 21, 46]. Zhou et al. [25] stated that while squid-based protein hydrolysate is a good nutrition source, incorporating it into plant-based diets does not improve the growth of white shrimp. In contrast, a recent study suggests that supplementing 20 ppm protein hydrolysate for *P. vannamei* to reduce feed by 30% has resulted in improved growth, total biomass, and survival rate [19].

The survival rates of juvenile lobsters in this study fall within the average survival rates of juvenile lobsters reared under laboratory conditions with an individual housing system [4, 35, 36]. While individual housing provides unlimited access to food for juvenile lobsters due to zero competition for food, this system sparks concerns about the stressful condition of housed animals, and thus requires further investigation. The spiny lobsters are ontogenic shifters with specific foraging habitats in every stage of life [47], which is critical in foraging and growing. In contrast, the communal system is close to lobsters' natural environment, enabling social interaction and stimulation of feeding habit that result in a higher growth rate than the individual system [48]. However, the communal system also allows cannibalism among lobsters, particularly during the intermolt period when their shells have not completely developed and their fitness is suboptimum due to the molting process. In this study, each aquarium is equipped with lobster shelters as recommended [49] to ensure that lobster mortality is not caused by the cannibalism but rather by nutritional factors.

The molting rate of juvenile lobsters in this study (62.50%–83.33%) was slightly higher than that of earlier study [35], which reported that the highest molting rate was achieved by juvenile lobsters fed with 35% of fishmeal replaced with DBSF meal. Molting is a critical growth process for the crustaceans in which the old exoskeleton is replaced with the new one in a process that, according to Lemos and Weissman [50], requires an immense energy to complete thorough molting and determine further development. The molting rate and molting period of crustaceans are strongly associated with nutrition requirements or other external factors, such as temperature [51]. In this study, not all juvenile lobsters complete the molting process despite having the same molting rate across treatments. The incomplete molting process that is observed to have occurred during the feeding trial has resulted in mortality and is the major contributor to the mortality of juvenile lobsters. Feed quality may be responsible for incomplete molting process because crustaceans require high energy to complete the molting process or ecdysis. In addition, this study has observed that low molting rate coincides with the low growth rate of the lobster, and thus requires further investigation.

The histopathology of animal organs reflects the animal's general condition and has been widely used to assess crustacean's health status [52, 53, 54, 55, 56] or the development of reproduction organ [57]. The present study has observed noticeable changes in the midgut and hepatopancreas structure of juvenile lobsters. The midgut villus—the most important part of the digestive system—has increased in size after the juvenile lobsters consumed FPH supplementation. In a previous study, the histopathology of hepatopancreas of juvenile lobsters was affected by the increased supplementation of DBSF meal, in which supplementation higher than 35% would cause damage in the species [35]. Another study reported a change in intestine structure of fish following BSF meal supplemented in the diet [58]. Although BSF meal is known as a good source of nutrition for animal feed [59, 60], it is scarcely used in the formulated aquafeeds [58, 61, 62, 63], and therefore, leaving the mechanism of nutrition absorption of the BSF meal in cultured fish and crustaceans in the unknown. One of the problems with the use of BSF meal in aquafeeds identified in this study is the high ash content. High ash in BSF was reportedly due to a high mineral composition [64]. Previous studies reported the apparent histological damage of the intestine following the excessive supplementation of BSF meal and proved the need to limit BSF utilization [35, 58]. The supplementation of chitinase reportedly improved the nutrition of BSF meal in the aquafeed [65]. In this study, the single use of FPH may not affect the growth and immunology status of juvenile lobsters. However, Halim et al. [66] reported that FPH supplemented into feed containing FBSF and DBSF could improve nutrient absorption in *P. vannamei* due to their biological function as antimicrobial peptides, which resulted in greater growth and feed intake of *P. vannamei* compared to animals not consuming FPH. Similarly, shrimp's digestive enzyme increased with the level of FPH supplementation in the diet, leading to higher abundance and diversity of phytoplankton and zooplankton in the shrimp ponds [41]. Both planktons are beneficial to foster optimum conditions for shrimp to grow by maintaining water quality in the shrimp pond and providing additional nutritional sources for penaeid shrimp post larvae [19].

The BSF meal is not only a protein source but also a lipid source in aquafeeds [18, 67, 68]. Research has shown that the Eicosapentaenoic acid (EPA) and docosahexaenoic acid (DHA) in the fish body are significantly affected by the inclusion of BSL meal in the diets [67] but BSF oil has no influence on the lipid class in the body composition of *Lates calcalifer* [18]. In this study, although DHA and EPA were found in the experimental feeds, the proximate composition of juvenile lobsters' whole body indicated the absence of DHA and EPA. The ∑SFA in the juvenile lobsters' whole body was not influenced by BSF meal without FPH supplementation, and there was a change in total unsaturated fat, ∑PUFA, ∑MUFA, ∑*n*−6 FA, and ∑*n*−9 FA. This study also indicates that FPH as a fishmeal substitute does not influence the general fatty acid profile of the juvenile lobster's whole body. FPH supplementation (particularly liquid) has an equivalent result with feeds containing FBSF and DBSF, namely reduced fatty acids in the body composition. Similarly, the increase of FPH supplementation in feeds significantly reduced the amount of total fat in the liver, digestive tract, and muscle in *O. pabda* [69]. Conversely, it increases the amount of total protein in the present study.

## 5. Conclusion

The supplementation of FPH in the feed containing FBSF and DBSF as the main protein sources has resulted in improved growth of juvenile lobsters. FPH increased nutrient absorption in feed to provide optimum nutrition for the juvenile lobsters to grow. However, the histopathological observations on the hepatopancreas and midgut of juvenile lobsters indicate that FPH supplementation does not improve the height of villus and muscle thickness of the intestine. The future feeding experiments on the lobster should consider undertaking additional analysis such as gut microbiome to provide further significant support to the present findings.

## Figures and Tables

**Table 1:** Experimental feed ingredients (g kg^−1^) dry weight basis.

Ingredients	Feed					
	T1	T2	T3	T4	T5	T6
Fishmeal	640	576	522	545	502	508
Corn starch	30	15	30	25	10	10
Wheat	119	68	122	123	66	60
Astaxanthin	2	2	2	2	2	2
Cholesterol	4	4	4	4	4	4
Fish oil	28	28	3	20	25	5
Soy lecithin	5	5	3	3	3	3
Vitamin premix	10	10	10	10	10	10
Mineral premix	5	5	5	5	5	5
Binder	32	22	32	22	22	22
Vitamin C	5	5	5	5	5	5
Casein	120	118	130	102	96	120
FPH	0	142	0	0	128	128
FBSF	0	0	132	0	0	118
DBSF	0	0	0	134	122	0
Supplementation (%)	0	24.7	25.3	24.6	24.3	25.2
Proximate composition						
Dry matter (%)	91.47	78.66	90.88	91.86	80.23	79.46
Ash (%)	8.52	8.47	8.13	8.62	8.65	8.44
Gross energy (MJ kg^−1^)^†^	18.07	17.70	18.09	18.32	17.71	17.77
Crude protein (%)	47.93	47.27	47.49	48.31	47.51	47.92
Lipid (%)	7.07	7.21	8.86	7.80	7.39	8.85
Protein-to-energy ratio (mg kJ^−1^)^†^	26.52	26.71	26.25	26.38	26.83	26.96

^†^Data were collected from the feed formulation software. *Note*. Fishmeal, from *Nemipterus virgatus* (defatted); crude protein 56%, crude fat 12 %, ash 11.8%. FPH, Propevia®; crude protein 25.6%, crude fat 4.0%, moisture 57.98%, ash 5.6%. FBSF, crude protein 40%; crude fat 39.7%, carbohydrate 11.4%, ash 10.97%. DBSF, crude protein 55%; crude fat 9%, carbohydrate 6%, and ash 10%.

**Table 2:** The fatty acids composition of full-fat black soldier fly (FBSF) and defatted black soldier fly (DBSF) meal.

Fatty acids	BSF (g kg^−1^)	
	DBSF	FBSF
Caprylic acid	ND	ND
Capric acid	ND	ND
Lauric acid	0.123	0.721
Myristic acid	0.752	1.023
Palmitic acid	1.523	2.645
Stearic acid	0.185	0.412
Arachidic acid	—	0.023
Oleic acid	1.712	2.345
Linoleic acid	2.612	3.432
Linolenic acid	0.105	0.212
Polyunsaturated fatty acids	2.717	3.667
Monounsaturated fatty acids	1.817	2.557
Saturated fatty acids	2.583	4.801

**Table 3:** The amino acids of fishmeal, defatted black soldier fly (DBSF), and full-fat black soldier fly (FBSF) meal.

Amino acid	Protein sources (g kg^−1^)		
	FM	DBSF	FBSF
Aspartate	0.243	0.184	0.235
Glutamine	0.443	0.312	0.321
Serine	0.081	0.071	0.165
Glycine	0.137	0.084	0.185
Histidine	0.098	0.072	0.043
Arginine	0.092	0.07	0.123
Threonine	0.119	0.082	0.077
Alanine	0.09	0.072	0.128
Proline	0.12	0.084	0.182
Tyrosine	0.093	0.071	0.078
Valine	0.079	0.061	0.127
Methionine	0.069	0.052	0.061
Cysteine	0.072	0.062	0.074
Isoleucine	0.118	0.088	0.118
Leucine	0.224	0.13	0.213
Phenylalanine	0.083	0.069	0.088
Lysine	0.179	0.126	0.216

**Table 4:** Production performances of juvenile lobsters.

Parameter	Feeds					
	T1	T2	T3	T4	T5	T6
SR	50.0 ± 0.07	45.8 ± 0.04	54.2 ± 0.15	50.0 ± 0.07	62.5 ± 0.07	66.7 ± 0.04
Final BW	0.43 ± 0.03^a^	0.42 ± 0.01^a^	0.41 ± 0.01^a^	0.43 ± 0.03^a^	0.46 ± 0.02^a,b^	0.51 ± 0.02^b^
Final TL	25.53 ± 0.32^a^	26.72 ± 0.23^a,b^	27.70 ± 0.95^b^	25.51 ± 0.17^a^	28.03 ± 0.95^b^	28.21 ± 0.48^b^
SGR	1.29 ± 0.05^a,b^	1.29 ± 0.03^a,b^	1.24 ± 0.03^a^	1.32 ± 0.08^a,b^	1.41 ± 0.03^a,b^	1.51 ± 0.05^b^
LI	24.16 ± 0.01^a^	32.75 ± 0.01^a,b^	35.16 ± 0.04^b^	22.33 ± 0.01^a^	36.69 ± 0.03^b^	38.12 ± 0.01^b^
MR	70.83 ± 0.11	62.50 ± 0.13	75.00 ± 0.12	83.33 ± 0.04	75.00 ± 0.12	79.17 ± 0.04

Initial mean weight was 0.21 ± 0.01 g and the mean total length was 20.53 ± 0.12 mm. Data are presented in the mean ± SE from three replicates. SR, survival rate (%); BW, wet body weight (g); TL, total length (mm); SGR, specific growth rate (% g/day); LI, length increment (%); MR, molting rate (%). Values bearing different superscripts within columns (a and b) represent a significant difference at *P*  < 0.05.

**Table 5:** Nutritional composition of juvenile lobsters' whole body (percentage of wet weight) is presented as mean ± SE.

Parameter	Feeds					
	T1	T2	T3	T4	T5	T6
Ash	11.15 ± 0.07^a^	11.22 ± 0.09^a^	15.60 ± 0.12^d^	11.62 ± 0.09^b^	11.22 ± 0.09^a^	12.36 ± 0.11^c^
Moisture	77.89 ± 0.22^d^	72.4 ± 0.21^b^	73.02 ± 0.13^b^	79.08 ± 0.10^e^	76.15 ± 0.18^c^	69.17 ± 0.29^a^
Carbohydrate	1.24 ± 0.21^a^	3.02 ± 0.06^d^	1.24 ± 0.01^a^	1.82 ± 0.01^b^	2.04 ± 0.02^c^	1.27 ± 0.01^a^
Fat	0.25 ± 0.01^b^	0.24 ± 0.01 ^a,b^	0.45 ± 0.01^d^	0.26 ± 0.01^b,c^	0.28 ± 0.01^c^	0.22 ± 0.01^a^
Protein	9.48 ± 0.26^b^	13.13 ± 0.35^c^	9.71 ± 0.23^b^	7.23 ± 0.20^a^	10.33 ± 0.26^b^	16.99 ± 0.39^d^

Values bearing different superscripts within columns (a, b, c, d, and e) represent a significant difference at *P*  < 0.05.

**Table 6:** Fatty acids composition of the wet weight of tested feeds (g kg^−1^) (mean ± SE).

Fatty acids	Feed					
	T1	T2	T3	T4	T5	T6
Arachidonic acid	0.03 ± 0.00^a^	0.05 ± 0.00^d^	0.13 ± 0.00^b^	0.05 ± 0.00^b^	0.07 ± 0.00^c^	0.05 ± 0.00^b^
Capric acid	0.01 ± 0.00	ND	ND	ND	ND	ND
Lauric acid	0.01 ± 0.00^a^	0.83 ± 0.01^f^	0.17 ± 0.00^b^	0.29 ± 0.01^c^	0.34 ± 0.00^d^	0.60 ± 0.00^e^
Myristic acid	0.14 ± 0.00^a^	0.37 ± 0.01^d^	0.58 ± 0.01^e^	0.23 ± 0.00^b^	0.24 ± 0.00^b^	0.29 ± 0.01^c^
Pentadecanoic acid	0.02 ± 0.00^a^	0.04 ± 0.00^a^	0.12 ± 0.00^e^	0.05 ± 0.00^c^	0.05 ± 0.00^d^	0.05 ± 0.00^c^
Palmitic acid	1.01 ± 0.01^a^	1.41 ± 0.01^b^	3.17 ± 0.00^d^	1.46 ± 0.01^b^	1.58 ± 0.01^c^	1.41 ± 0.03^b^
Palmitoleic acid	0.17 ± 0.00^a^	0.24 ± 0.01^b^	0.57 ± 0.01^d^	0.23 ± 0.00^b^	0.27 ± 0.00^c^	0.22 ± 0.00^b^
Heptadecanoic acid 17 : 0	0.04 ± 0.00^a^	0.06 ± 0.00^b^	0.18 ± 0.00^c^	0.06 ± 0.00^b^	0.08 ± 0.00^c^	0.06 ± 0.00^b^
Heptadecanoic acid 17 : 1	0.02 ± 0.00^a^	0.03 ± 0.00^c^	0.08 ± 0.00^d^	0.03 ± 0.00^c^	0.03 ± 0.00^c^	0.03 ± 0.00^b^
Stearic acid	0.36 ± 0.01^a^	0.44 ± 0.01^b^	1.11 ± 0.02^e^	0.51 ± 0.01^cd^	0.52 ± 0.01^d^	0.47 ± 0.00^bc^
C Oleic acid	2.55 ± 0.03^c^	1.81 ± 0.02^a^	4.67 ± 0.02^d^	1.93 ± 0.02^a^	2.43 ± 0.07^c^	2.21 ± 0.01^b^
C Linoleic acid	1.75 ± 0.02^e^	0.77 ± 0.02^a^	2.16 ± 0.01^f^	1.01 ± 0.01^c^	1.20 ± 0.02^d^	0.87 ± 0.02^b^
Linolenic acid, *ω*6	0.01 ± 0.00	ND	ND	ND	ND	ND
Linolenic acid, *ω*3	0.30 ± 0.01^c^	0.10 ± 0.00^a^	0.38 ± 0.01^d^	0.10 ± 0.00^a^	0.16 ± 0.00^b^	0.10 ± 0.00^a^
Arachidic acid	0.03 ± 0.00	ND	0.06 ± 0.00	ND	ND	ND
Eicocyanic acid	0.12 ± 0.00^e^	0.05 ± 0.00^c^	0.20 ± 0.00^f^	0.04 ± 0.00^b^	0.06 ± 0.00^d^	0.02 ± 0.00^a^
Eicosadienoic acid	0.06 ± 0.00	ND	0.09 ± 0.00	ND	ND	ND
Eicosatrienoic acid, *ω*3	0.02 ± 0.00	ND	ND	ND	ND	ND
Eicosatrienoic acid, *ω*6	0.02 ± 0.00	ND	ND	ND	ND	ND
Arachidonic acid	0.03 ± 0.00^a^	0.05 ± 0.00^b^	0.13 ± 0.00^d^	0.05 ± 0.00^b^	0.07 ± 0.00^c^	0.05 ± 0.00^b^
Eicosatpentaenoic acid	0.20 ± 0.00^e^	0.10 ± 0.00^c^	0.48 ± 0.00^f^	0.07 ± 0.00^a^	0.13 ± 0.00^d^	0.08 ± 0.00^b^
Erucic acid	0.01 ± 0.00	ND	ND	ND	ND	ND
Docosahexanoic acid	0.27 ± 0.00^b^	0.25 ± 0.00^ab^	1.03 ± 0.01^d^	0.23 ± 0.01^a^	0.31 ± 0.01^c^	0.25 ± 0.01^ab^
DHA	0.27 ± 0.00^b^	0.25 ± 0.00^ab^	1.03 ± 0.01^d^	0.23 ± 0.01^a^	0.31 ± 0.01^c^	0.25 ± 0.01^ab^
EPA	0.20 ± 0.00^e^	0.10 ± 0.00^c^	0.48 ± 0.00^f^	0.07 ± 0.00^a^	0.13 ± 0.00^d^	0.08 ± 0.00^b^
Unsaturated fat	5.53 ± 0.07^d^	3.40 ± 0.00^a^	9.80 ± 0.02^e^	3.69 ± 0.00^b^	4.66 ± 0.05^c^	3.83 ± 0.02^b^
∑SFA	1.60 ± 0.02^a^	3.15 ± 0.04^d^	5.38 ± 0.01^e^	2.59 ± 0.03^b^	2.82 ± 0.01^c^	2.88 ± 0.04^c^
∑PUFA	2.66 ± 0.03^d^	1.27 ± 0.01^a^	4.28 ± 0.01^e^	1.46 ± 0.02^b^	1.86 ± 0.03^c^	1.35 ± 0.01^a^
∑MUFA	2.86 ± 0.04^c^	2.13 ± 0.01^a^	5.5 ± 0.02^d^	2.23 ± 0.02^a^	2.80 ± 0.08^c^	2.48 ± 0.01^b^
∑*n*−3 FA	0.79 ± 0.01^d^	0.45 ± 0.00^b^	1.90 ± 0.00^e^	0.40 ± 0.01^a^	0.60 ± 0.01^c^	0.43 ± 0.01^ab^
∑*n*−6 FA	1.81 ± 0.02^e^	0.82 ± 0.01^a^	2.29 ± 0.01^f^	1.06 ± 0.01^c^	1.27 ± 0.01^d^	0.92 ± 0.02^b^
∑*n*−9 FA	2.56 ± 0.03^c^	1.81 ± 0.02^a^	4.67 ± 0.02^d^	1.93 ± 0.02^a^	2.43 ± 0.07^c^	2.21 ± 0.01^b^
Linoleic acid, *ω*6	1.75 ± 0.02^e^	0.77 ± 0.01^a^	2.16 ± 0.01^f^	1.01 ± 0.01^c^	1.20 ± 0.02^d^	0.87 ± 0.02^b^
Oleic acid	2.55 ± 0.03^c^	1.81 ± 0.02^a^	4.67 ± 0.02^d^	1.93 ± 0.02^a^	2.43 ± 0.07^c^	2.21 ± 0.01^b^
Linolenic acid	0.31 ± 0.01^c^	0.10 ± 0.00^a^	0.38 ± 0.01^d^	0.10 ± 0.00^a^	0.16 ± 0.00^b^	0.10 ± 0.00^a^
Linoleic acid	1.75 ± 0.02^e^	0.77 ± 0.01^a^	2.16 ± 0.01^f^	1.01 ± 0.01^c^	1.20 ± 0.02^d^	0.87 ± 0.02^b^

ND, not detected. Values bearing different superscripts within columns (a, b, c, d, e, and f) represent a significant difference at *P* < 0.05.

**Table 7:** Fatty acids composition of juvenile lobsters' whole body (g kg^−1^) wet weight (mean ± SE).

Fatty Acids	Feeds					
	T1	T2	T3	T4	T5	T6
Palmitic acid	0.84 ± 0.03^b^	0.76 ± 0.02^ab^	1.30 ± 0.03^d^	0.73 ± 0.02^a^	0.99 ± 0.03^c^	0.77 ± 0.03^ab^
Palmitoleic acid	0.20 ± 0.00	ND	0.42 ± 0.00	0.30 ± 0.01	ND	ND
Stearic acid	0.36 ± 0.0^b^	0.30 ± 0.00^ab^	0.52 ± 0.00^d^	0.37 ± 0.01^a^	0.36 ± 0.01^c^	0.32 ± 0.01^ab^
C Oleic acid	0.69 ± 0.02^a^	0.91 ± 0.03^b^	1.37 ± 0.02^d^	0.76 ± 0.02^a^	1.02 ± 0.02^c^	0.70 ± 0.02^a^
C Linoleic acid	0.36 ± 0.01^a^	0.39 ± 0.01^a^	0.84 ± 0.02^b^	0.39 ± 0.01^a^	0.38 ± 0.00^a^	0.37 ± 0.01^a^
Unsaturated fat	1.25 ± 0.03^b^	1.30 ± 0.03^b^	2.63 ± 0.05^d^	1.45 ± 0.04^c^	1.40 ± 0.01^bc^	1.06 ± 0.03^a^
∑SFA	1.20 ± 0.04^b^	1.05 ± 0.03^a^	1.82 ± 0.03^d^	1.10 ± 0.00^ab^	1.35 ± 0.04^c^	1.09 ± 0.04^ab^
∑PUFA	0.36 ± 0.0^a^	0.39 ± 0.04^a^	0.84 ± 0.02 ^b^	0.39 ± 0.0^a^	0.38 ± 0.00^a^	0.37 ± 0.01^a^
∑MUFA	0.89 ± 0.02^b^	0.91 ± 0.03^b^	1.79 ± 0.02^d^	1.06 ± 0.03^c^	1.02 ± 0.02^c^	0.70 ± 0.02^a^
∑*n*−6 FA	0.36 ± 0.01^a^	0.39 ± 0.01^a^	0.84 ± 0.02^b^	0.39 ± 0.01^a^	0.38 ± 0.00^a^	0.37 ± 0.01^a^
∑*n*−9 FA	0.70 ± 0.02^a^	0.91 ± 0.03^b^	1.37 ± 0.02^d^	0.76 ± 0.02^a^	1.02 ± 0.02^c^	0.70 ± 0.02^a^
Linoleic acid	0.36 ± 0.01^a^	0.39 ± 0.01^a^	0.84 ± 0.02^b^	0.39 ± 0.01^a^	0.38 ± 0.00^a^	0.37 ± 0.01^a^
Oleic acid	0.70 ± 0.02^a^	0.91 ± 0.03^b^	1.37 ± 0.02^d^	0.76 ± 0.02^a^	1.02 ± 0.02^c^	0.70 ± 0.02^a^

Data in the similar column with different superscripts (a, b, c, and d) represent a significant difference at *P*  < 0.05. ND, not detected.

## Data Availability

The data used to support the findings of this study are available from the corresponding author upon request
